# Effective Use of Water and Increased Dry Matter Partitioned to Grain Contribute to Yield of Common Bean Improved for Drought Resistance

**DOI:** 10.3389/fpls.2016.00660

**Published:** 2016-05-12

**Authors:** Jose A. Polania, Charlotte Poschenrieder, Stephen Beebe, Idupulapati M. Rao

**Affiliations:** ^1^Centro Internacional de Agricultura TropicalSantiago de Cali, Colombia; ^2^Lab Fisiología Vegetal, Facultad de Biociencias, Universidad Autónoma de BarcelonaBellaterra, Spain

**Keywords:** canopy biomass, carbon isotope discrimination, pod harvest index, terminal drought stress, water use efficiency

## Abstract

Common bean (*Phaseolus vulgaris* L.) is the most important food legume in the diet of poor people in the tropics. Drought causes severe yield loss in this crop. Identification of traits associated with drought resistance contributes to improving the process of generating bean genotypes adapted to these conditions. Field studies were conducted at the International Center for Tropical Agriculture (CIAT), Palmira, Colombia, to determine the relationship between grain yield and different parameters such as effective use of water (EUW), canopy biomass, and dry partitioning indices (pod partitioning index, harvest index, and pod harvest index) in elite lines selected for drought resistance over the past decade. Carbon isotope discrimination (CID) was used for estimation of water use efficiency (WUE). The main objectives were: (i) to identify specific morpho-physiological traits that contribute to improved resistance to drought in lines developed over several cycles of breeding and that could be useful as selection criteria in breeding; and (ii) to identify genotypes with desirable traits that could serve as parents in the corresponding breeding programs. A set of 36 bean genotypes belonging to the Middle American gene pool were evaluated under field conditions with two levels of water supply (irrigated and drought) over two seasons. Eight bean lines (NCB 280, NCB 226, SEN 56, SCR 2, SCR 16, SMC 141, RCB 593, and BFS 67) were identified as resistant to drought stress. Resistance to terminal drought stress was positively associated with EUW combined with increased dry matter partitioned to pod and seed production and negatively associated with days to flowering and days to physiological maturity. Differences in genotypic response were observed between grain CID and grain yield under irrigated and drought stress. Based on phenotypic differences in CID, leaf stomatal conductance, canopy biomass, and grain yield under drought stress, the lines tested were classified into two groups, water savers and water spenders. Pod harvest index could be a useful selection criterion in breeding programs to select for drought resistance in common bean.

## Introduction

Common bean (*Phaseolus vulgaris* L.) is the most important food legume in the tropics of Latin America and eastern and southern Africa. Beans are predominantly cultivated by small farmers in these two regions where climate variability and limited use of inputs frequently reduce productivity (Beebe, 2012; Beebe et al., 2013). The yield of beans is affected by various constraints (Thung and Rao, 1999). Among those drought is responsible for losses between 10 and 100%. About 60% of the bean-producing regions have prolonged periods of water shortage and drought is the second most important factor in yield reduction after diseases (Thung and Rao, 1999; Rao, 2014). The development of bean varieties resistant to drought stress conditions through breeding is a useful strategy to increase food security in marginal areas. Breeding programs for improving resistance to drought usually select the best genotypes based on grain yield under drought stress (Rosales et al., 2012). A physiological approach using morpho-physiological traits as selection criteria can increase the possibility of combining parents with complementary traits, provided the germplasm is characterized more thoroughly than just testing for yield (Reynolds and Trethowan, 2007; Mir et al., 2012). A useful trait must exhibit higher heritability, enough genetic variability, correlation with yield, and its evaluation must be fast, easy and cheap (Jackson et al., 1996; Araus et al., 2002; Beebe et al., 2013).

Three key processes, among others, have been related to improved drought resistance: (i) acquiring greater amount of water by the root system from the soil profile to facilitate transpiration, (ii) acquiring more carbon (biomass) in relation to water transpired, and (iii) increased mobilization of accumulated carbon to the harvestable economic product (Condon et al., 2004). Previous research identified several traits that contribute to improved resistance of common bean to drought and these include earliness, deep rooting, and greater ability to partition dry matter to grain production (Hall, 2004; Beebe et al., 2013; Rao et al., 2013; Rao, 2014). Water use efficiency (WUE), or “more crop per drop” is considered as an important component of drought resistance in different crops (Blum, 2009; Sinclair, 2012; Vadez et al., 2014). Increased WUE reduces the rate of transpiration and crop water use, processes that are crucial for carbon assimilation, biomass production, and yield (Blum, 2009; Sinclair, 2012). However, the reduction in water use is generally achieved by plant traits and environmental responses that could also reduce yield potential (Blum, 2005). WUE is a complex trait and difficult to phenotype, preventing many breeding programs from using WUE directly (Araus et al., 2002; Easlon et al., 2014). In contrast to WUE, effective use of water (EUW) implies maximal soil moisture capture for transpiration, and also involves decreased non-stomatal transpiration and minimal water loss by soil evaporation (Blum, 2009). EUW is relevant when there is still soil water available at maturity or when deep-rooted genotypes access water deep in the soil profile that is not normally available (Araus et al., 2002). Ideotypes of plants have been proposed by Blum (2015) for targeting in plant breeding according to agro-ecological zones and types of drought such as, the isohydric (“water saving”) plant model and the anisohydric (“water spending”) plant model. The water saving model might have an advantage in the harshest environments, whereas the water spending model will perform relatively better under more moderate drought conditions.

One plant attribute that has been associated with WUE is carbon isotope discrimination (CID), based on the inverse relationship between CID and WUE (greater ^13^C discrimination being associated with lower values of WUE, or conversely, more water use and transpiration). Selection for low ^13^C discrimination has been proposed as a screening method to improve WUE in breeding C_3_ crops (Araus et al., 2002; Khan et al., 2007; Easlon et al., 2014). Use of CID presented some advantages by reflecting integration over long periods of gas exchange during crop development, high throughput sampling, relatively low cost, and high heritability (Easlon et al., 2014). In bush bean under moderate droughts or non-arid environments, it has been observed that there is a positive relationship between CID, root length density and grain yield (Sponchiado et al., 1989; White et al., 1990; White, 1993; Hall, 2004; Polania et al., 2012). Increased water use is associated with increased accumulation of carbon and plant growth. However, improved harvest index (HI) or enhanced mobilization of photosynthates to grain production plays an essential role in increase of yield under stress. In most environments with drought, water deficit occurs during reproductive development, reducing HI (Blum, 2009). Several studies in common bean have shown that increased dry matter partitioned to pod and seed formation contributes to better grain yield under drought as well as low soil fertility stress (Rao, 2014). Therefore, assimilate partitioning is an important attribute to evaluate adaptation to abiotic stress in common bean (Rosales-Serna et al., 2004; Beebe et al., 2008, 2013; Klaedtke et al., 2012; Polania et al., 2012, 2016; Rosales et al., 2012; Assefa et al., 2013; Rao et al., 2013; Rao, 2014).

The main objectives of this study were: (i) to identify specific morpho-physiological traits that contribute to improved resistance to drought in lines developed over several cycles of breeding and that could be useful as selection criteria in breeding beans for drought resistance; and (ii) to identify genotypes with desirable traits that could serve as parents in breeding programs that are aimed to improve drought resistance.

## Materials and methods

### Experimental site and meteorological conditions

Two field trials were conducted during the dry season (from June to September in both 2012 and 2013), at the main experiment station of the International Center for Tropical Agriculture (CIAT) in Palmira, Colombia, located at 3° 29″ N latitude, 76° 21″ W longitude and at an altitude of 965 m. Basic characteristics of this field site were described previously (Beebe et al., 2008). The soil is a Mollisol (Aquic Hapludoll) with adequate nutrient supply and is estimated to permit storage of 100 mm of available water (assuming 1.0 m of effective root growth with −0.03 and −1.5 MPa as upper and lower limits for soil matric potential). During the crop-growing season, maximum and minimum air temperatures in 2012 were 31.0 and 19.0°C, and in 2013 were 30.2 and 19.2°C, respectively. Total rainfall during the active crop growth was 85.8 mm in 2012 and 87.7 mm in 2013. The potential pan evaporation was of 385.2 mm in 2012 and 351.0 mm in 2013. Two levels of water supply (irrigated and rainfed) were applied to simulate well-watered (control) and drought stress treatments respectively. Trials were furrow irrigated (~35 mm of water per irrigation). The drought stress treatment under rainfed conditions in 2012 received three irrigations (at 3 days before planting and at 5 and 23 days after planting) and in 2013 also received three irrigations (at 3 days before planting and at 4 and 15 days after planting). In both years, irrigation was suspended after the third irrigation to induce terminal drought stress conditions. The irrigated control treatment received five irrigations in 2012 and six irrigations in 2013 to ensure adequate soil moisture for crop growth and development.

### Plant material and experimental design

For this study 36 bush bean genotypes belonging to the Middle American gene pool were selected: 22 elite lines of common bean (BFS 10, BFS 29, BFS 32, BFS 67, MIB 778, NCB 226, NCB 280, RCB 273, RCB 593, SCR 16, SCR 2, SCR 9, SEN 56, SER 118, SER 119, SER 125, SER 16, SER 48, SER 78, SMC 141, SMC 43, and SXB 412); five interspecific lines between elite line SER 16 and *Phaseolus coccineus* (ALB 6, ALB 60, ALB 74, ALB 88, and ALB 213); one landrace of tepary bean (*Phaseolus acutifolius*) G40001 from Veracruz-Mexico, and two interspecific lines between tepary bean and common bean (INB 841 and INB 827 developed from five cycles of congruity backcrossing of tepary with ICA Pijao). BFS (small red) lines were developed to improve adaptation to low soil fertility and drought. SER and SCR (small red), SEN (small black), and NCB (small black) lines were developed for improved adaptation to drought. ALB (small red) lines were developed for improved adaptation to drought and aluminum toxicity. RCB (small red) lines were developed for improved yield potential, disease resistance, and commercial grain. SEA 15 and BAT 477 were included as drought resistant checks, and three commercial cultivars of common bean (DOR 390, Pérola and Tio Canela) as drought sensitive materials. BAT 477 NN was included as a non-nodulating bean genotype. In the 2 years, a 6 × 6 partially balanced lattice design with three replications was used. Experimental units consisted of 4 rows with 3.72 m row length with a row-to-row distance of 0.6 m and plant-to-plant spacing of 7 cm (equivalent to 24 plants m^−2^). Trials were managed by controlling weeds with application of herbicides (Fomesafen, Fluazifop-p-butil, and Bentazon) and pests and diseases by spraying with insecticides (Thiametoxam, Clorpirifos, Imidacloprid, Abamectina, Cyromazine, and Milbemectin) and fungicides (Benomil and Carboxin) as needed.

### Yield measurements and phenological assessment

Grain was harvested from two central rows after discarding end plants in both the irrigated and drought plots. Mean yields per hectare were corrected for 0% moisture in grain. Days to flowering (DF) and days to physiological maturity (DPM) were recorded for each plot. DF is defined as the number of days after planting until 50% of the plants have at least one open flower. DPM is the number of days after planting until 50% of plants have at least one pod losing its green pigmentation. The geometric mean (GM) of grain yield was determined as (NS × DS)^1∕2^ where NS is no stress (irrigated) and DS is drought stress. The drought response index (DRI) was calculated as indicator of drought resistance as described before (Bidinger et al., 1987; Krishnamurthy et al., 2010), using DF under rainfed conditions for every individual plot and yield potential as arithmetic mean of grain yield under irrigated conditions across replications.

### Physiological measurements

At mid-pod filling, a 50 cm segment of the row (equivalent to an area of 0.3 m^2^) from each plot with about seven plants was used for destructive sampling to measure total canopy biomass (CB) and leaf area index (LAI). Leaf area was measured using a leaf area meter (model LI-3000, LI-COR, NE, USA) and the LAI was calculated as leaf area per unit land area. Also, at mid-pod filling the stomatal conductance was measured with a portable leaf porometer (Decagon SC-1) on a fully expanded young leaf of one plant within each replication. Measurements were made late in the morning $(10 a.m.–12 noon) on clear, sunny days with minimal wind. At the time of harvest, plants in 50 cm of a row from each plot were cut and dry weights of stem, pod, seed, and pod wall, seed number per area (SNA), and pod number per area (PNA) were recorded. The following attributes were determined according to Beebe et al. (2013): harvest index (HI) (%): seed biomass dry weight at harvest/total shoot biomass dry weight at mid-pod filling × 100; pod harvest index (PHI) (%): seed biomass dry weight at harvest/pod biomass dry weight at harvest × 100; pod partitioning index (PPI) (%): pod biomass dry weight at harvest/total canopy biomass dry weight at mid-pod filling × 100. HI and PPI were estimated using the CB value at mid-pod filling growth stage which is assumed to be the time that reflects the maximum vigor of the genotype. It is assumed that from this time common bean begins to lose canopy biomass through leaf fall, especially under terminal drought stress. Shoot and seed total nonstructural carbohydrates (TNC) concentrations were determined according to the method described by Kang and Bringk (1995).

One plant of each genotype from each plot (irrigated and drought) was selected for destructive sampling at mid-pod filling. A random sample of grain per experimental unit was selected, washed thoroughly and ground. The ground samples of plants at mid-pod filling and grain at harvest were sent to UC Davis Stable Isotope Facility in USA for ^13^C analysis. CID (Δ^13^C in ‰) was calculated according to the following equation, where δ^13^Cs and δ^13^Ca are sample and atmospheric concentrations of ^13^C, respectively, and carbon isotope composition of atmosphere is assumed to be −8.0‰ (Farquhar et al., 1989). Isotopic discrimination between ^13^C and ^12^C (Δ) in shoot and grain was related to whole plant water use (Farquhar et al., 1989).

$$\Delta \text{ 13C }(\text{CID})\text{ =}\frac{\left[\text{δ13Ca }-\text{ δ13Cs}\right]}{\left[\text{1 + }(\text{δ13Cs/1000})\right]}$$

### Statistical analysis

All data were analyzed using the SAS (v 9.0) PROC MIXED and PROC CORR (SAS Institute Inc., 2008). The adjusted means for each genotype and the environment (irrigated and rainfed) were obtained using the mixed models theory together with the MIXED procedure considering the effects of the replications and blocks within replications as random and genotypes as fixed. Correlation coefficients were calculated by the PROC CORR. Values reported with ^*^, ^**^, or ^***^ are statistically significant at probability levels of 5, 1, and 0.1%, respectively.

## Results

### Grain yield

The data on rainfall distribution, water applied through irrigation, and pan evaporation in both trials indicated that the crop suffered terminal drought stress during crop development under rainfed treatment conditions. The mean value of grain yield under drought conditions decreased by 56% compared with irrigated conditions (Table 1). Under drought conditions the grain yield of 36 genotypes ranged from 59 to 1526 kg ha^−1^ (Table 1). Among the genotypes tested, the lines BFS 29, NCB 280, SEN 56, BFS 10, SEA 15, and NCB 226 were superior in their resistance to drought stress. The relationship between grain yield of drought and irrigated treatments indicated that BFS 29, NCB 280, SEN 56, BFS 10, and NCB 226 were not only drought resistant but were also responsive to irrigation (Table 1). Among the 36 genotypes tested, the biofortified line MIB 778, was the most sensitive to drought stress. MIB 778 is an interspecific progeny of common bean and *P. dumosus*, which may explain its extreme sensitivity to drought. The genotypes Pérola, DOR 390, SMC 43, and ALB 88 were also sensitive to drought stress (Table 1).

**Table 1:** **Phenotypic differences in canopy biomass, pod partitioning index, grain yield and drought response index (DRI) of 36 genotypes of common bean grown under irrigated and drought conditions in 2012 and 2013 at Palmira, Colombia**.

**Genotype**	**Canopy biomass (kg ha**^**−1**^**)**		**Pod partitioning index (%)**		**Grain yield (kg ha**^**−1**^**)**			**DRI**
	**Irrigated**	**Drought**	**Irrigated**	**Drought**	**Irrigated**	**Drought**	**GM**	
NCB 280	4695	3165	87	74	2922	1457	2117	0.70
BFS 29	4391	3788	87	65	2971	1526	2012	0.31
NCB 226	3742	3051	101	69	2973	1316	2000	0.56
SEN 56	4988	3063	80	74	2898	1330	1996	0.54
BFS 10	4074	2944	84	66	2700	1409	1905	0.76
SCR 2	4554	3636	76	66	2495	1272	1887	0.61
SEA 15	4188	3377	92	70	2243	1451	1867	0.64
SER 118	4215	2367	70	94	2508	798	1816	0.35
ALB 74	4318	2871	80	55	2127	971	1776	0.06
ALB 6	4240	2727	91	68	2469	1006	1743	0.06
BFS 67	4763	2992	74	62	2662	1163	1739	0.57
RCB 593	4560	3329	74	52	2519	1321	1691	0.35
SER 48	4448	3455	81	57	2368	1046	1677	0.02
SMC 43	3967	2534	57	47	1682	442	1670	1.00
BFS 32	4525	2655	99	67	2918	1037	1670	−0.75
SCR 9	4161	2990	90	57	2312	1053	1656	−0.04
SER 125	4901	3710	66	59	2432	1178	1647	0.45
SER 78	4892	3374	61	73	2345	1116	1591	−0.34
SER 119	4228	3480	80	55	2459	1120	1572	−0.06
SCR 16	4709	2935	86	74	2744	1310	1536	−0.27
G 40001	3810	3073	71	52	2233	1035	1522	−0.31
INB 827	4847	3181	64	63	2247	1069	1512	0.40
RCB 273	4552	3025	90	57	2425	934	1497	−0.61
ALB 60	4671	2899	76	44	2669	1162	1488	−0.33
INB 841	3268	2408	112	61	2316	845	1447	−1.06
SXB 412	3814	2680	89	65	2507	937	1436	0.14
BAT 477	4501	2982	71	61	2131	983	1435	0.71
SER 16	4443	3694	84	43	2502	1252	1381	−0.27
ALB 88	3826	2346	65	53	2018	544	1334	−0.66
Tío Canela 75	4148	2401	70	78	1936	805	1219	0.21
ALB 213	4641	2874	72	57	2659	1262	1092	−0.90
SMC 141	3894	2298	82	87	2592	1189	939	−0.88
DOR 390	4411	2452	64	32	2259	505	871	−0.81
BAT 477 NN	2212	2382	99	44	1078	771	845	0.30
Pérola	3523	2120	87	49	1838	413	837	−0.39
MIB 778	4108	2102	60	15	828	59	288	−1.07
Mean	4256	2927	80	60	2361	1030	1520	0
Sig. diff.	^*^	^*^	NS	^*^	^*^	^*^	^*^	^*^

Values reported are mean for two seasons. GM = geometric mean.

* Significant difference at 0.05 level as estimated from the MIXED procedure.

### Leaf stomatal conductance and carbon isotope discrimination

Leaf stomatal conductance presented a significant positive correlation with grain yield under both irrigated (0.24^**^) and drought stress (0.31^***^) conditions (**Table 3**). The lines NCB 280, SEN 56, SCR 16, SMC 141, NCB 226, SEA 15, and BFS 10, combined higher values of leaf stomatal conductance with better grain yield under drought stress (Tables 1, 2). The lines MIB 778, Pérola, SMC 43, and DOR 390 presented lower values of stomatal conductance combined with lower grain yield under drought stress (Tables 1, 2). Similarly correlations and differences in genotypic response were observed between grain CID and grain yield under irrigated and drought stress conditions (Table 3). The accession of *P. acutifolius* G 40001 and the lines SER 16, ALB 6, and ALB 60 presented lower values of leaf stomatal conductance and grain CID (lower use of water) combined with moderate plant growth and grain yield under drought stress (Tables 1, 2).

**Table 2:** **Phenotypic differences in days to flowering, days to physiological maturity and leaf stomatal conductance of 36 genotypes of common bean grown under irrigated and drought conditions in 2012 and 2013 at Palmira, Colombia**.

**Genotype**	**Days to flowering**		**Days to physiological maturity**		**Leaf stomatal conductance (mmol m**^**−2**^ **s**^**−1**^**)**	
	**Irrigated**	**Drought**	**Irrigated**	**Drought**	**Irrigated**	**Drought**
ALB 6	34	34	60	62	351	317
ALB 60	32	32	56	57	309	342
ALB 74	33	33	59	59	354	398
ALB 88	34	35	61	62	375	441
ALB 213	32	33	59	60	361	366
BAT 477	36	37	62	61	265	357
BAT 477 NN	37	38	63	62	373	508
BFS 10	32	33	57	58	382	408
BFS 29	31	31	58	57	410	320
BFS 32	31	32	57	56	282	399
BFS 67	35	36	60	62	311	485
DOR 390	38	39	65	63	378	346
G 40001	32	32	55	55	288	345
INB 827	35	36	62	61	384	393
INB 841	31	30	57	55	305	258
MIB 778	39	40	66	67	351	277
NCB 226	31	33	64	62	331	442
NCB 280	30	31	56	57	417	629
Pérola	39	40	68	67	360	268
RCB 273	32	33	60	60	324	321
RCB 593	31	33	56	58	357	328
SCR 2	32	32	61	60	436	395
SCR 9	32	33	59	57	447	331
SCR 16	33	34	59	59	372	506
SEA 15	32	30	58	57	271	429
SEN 56	32	32	60	58	335	492
SER 16	31	32	56	57	383	310
SER 48	32	33	59	57	348	344
SER 78	34	35	59	59	300	363
SER 118	34	36	62	60	404	331
SER 119	33	33	59	59	364	361
SER 125	32	32	56	57	360	377
SMC 43	35	36	59	59	337	246
SMC 141	37	38	62	63	327	510
SXB 412	36	37	60	60	350	234
Tío Canela 75	37	38	64	64	315	348
Mean	34	34	60	60	350	376
Sig. diff.	^*^	^*^	^*^	^*^	NS	^*^

Values reported are mean for two seasons.

* Significant difference at 0.05 level as estimated from the MIXED procedure.

**Table 3:** **Correlation coefficients (r) between final grain yield (kg ha^**−1**^) and other shoot attributes of 36 genotypes of common bean grown under irrigated and drought stress conditions in 2012 and 2013 at Palmira, Colombia**.

**Plant traits**	**Irrigated**	**Drought**
Leaf area index (m^2^/m^2^)	0.12	0.43^***^
Canopy biomass (kg ha^−1^)	0.39^***^	0.59^***^
Leaf stomatal conductance (mmol m^−2^ s^−1^)	0.24^***^	0.31^***^
Pod partitioning index (%)	0.14^*^	0.29^***^
Harvest index (%)	0.24^***^	0.39^***^
Pod harvest index (%)	0.61^***^	0.48^***^
Shoot TNC content (mg g^−1^)	−0.21^**^	0.05
Seed TNC content (mg g^−1^)	0.21^**^	0.16^*^
Days to flowering	−0.51^***^	−0.53^***^
Days to physiological maturity	−0.37^***^	−0.36^***^
Pod number per area (no. m^−2^)	0.32^***^	0.55^***^
Seed number per area (no. m^−2^)	0.36^***^	0.63^***^
100 seed weight (g)	0.44^***^	0.25^***^
Shoot CID (‰)	−0.12	0.15^*^
Grain CID (‰)	0.37^***^	0.36^***^

Mean values of two seasons were used for correlation analysis.

*, **, *** Significant at the 0.05, 0.01, and 0.001 probability levels, respectively.

### Canopy biomass (CB), pod partitioning index (PPI), and pod harvest index (PHI)

A positive and significant correlation was observed between CB and grain yield under both irrigated and drought conditions, 0.39^***^ and 0.59^***^, respectively (Table 3). The drought resistant lines combined higher CB production with higher grain yield under drought stress conditions. The drought susceptible lines presented lower values of both CB and grain yield under drought conditions (Table 1). It is important to note that the sampled area to determine CB at mid-pod filling was small and this could overestimate the total CB values per hectare. This small area was selected to facilitate comparison of large number of genotypes. Lines with superior grain yield showed higher values of LAI under drought stress (Table 3). The PPI reflects the biomass partitioned to pods at harvest as a proportion of the total CB at mid-pod filling growth stage. This ratio and HI may be overestimated because we used the CB values at mid-pod filling growth stage with the assumption that this growth stage reflects the maximum vigor. The values of CB may be underestimated if vegetative growth continues between mid-pod filling to physiological maturity due to irrigation or intermittent rainfall. Correlation coefficients between grain yield and PPI were positive and highly significant under drought conditions (Table 3). Also, the drought resistant lines combined higher value of PPI and grain yield under drought stress conditions while the drought susceptible lines showed lower ability in partitioning of dry matter to pod production under drought conditions (Table 1). The lines SMC 141 and SER 118 were outstanding in dry matter partitioning to pod production, but the CB values of these lines were lower under drought stress (Table 1).

The PHI value reflects the biomass partitioned to seed as a proportion of total pod biomass. A positive and highly significant correlation of PHI with grain yield was observed under both irrigated and drought conditions (Table 3). The lines identified as drought resistant were superior in PHI, resulting in a higher grain yield under drought conditions. The drought susceptible lines described previously showed lower ability in partitioning of dry matter from plant structures to pod production (PPI) and from podwall to seed production (PHI) resulting in poor performance under drought stress (**Figure 2**, Table 1). The accession of *P. acutifolius* (G 40001) and the lines INB 841 and SER 118 likewise presented higher values of PHI under drought stress. A weak positive correlation between grain yield and seed TNC was observed under both irrigated and drought stress conditions (Table 3). The drought resistant lines combined higher seed TNC concentration with higher grain yield under drought stress. The drought susceptible lines presented lower seed TNC concentration and grain yield under drought stress.

### Days to flowering (DF), days to physiological maturity (DPM), pod number per area (PNA), seed number per area (SNA), and 100 seed weight

Results on the analysis of DRI showed that the lines SMC 141, BFS 10, BAT 477, NCB 280, SEA 15, SCR 16, BFS 67, NCB 226, SEN 56, and SER 78 were drought resistant with higher values of DRI, while Pérola, RCB 273, ALB 74, BFS 32, DOR 390, SMC 43, ALB 88, INB 841, and MIB 778 were highly drought sensitive with lower values of DRI (Table 1). A negative and significant correlation was observed between DF and grain yield under both irrigated and drought conditions, −0.51^***^ and −0.53^***^, respectively (Table 3). The lines identified as drought resistant described previously combined shorter DF with higher grain yield under drought stress conditions (Tables 1, 2). The drought susceptible checks Tío Canela, Pérola, and DOR 390 and the lines MIB 778 and SMC 43 showed sensitivity to drought stress with greater DF under drought conditions (Table 2). The same response was observed in DPM (Table 2), that showed a negative and highly significant correlation with grain yield under both irrigated and drought treatments (Table 3). The drought resistant lines with shorter DF also showed shorter DPM with superior grain yield than the other genotypes under drought stress (Tables 1, 2). A negative and significant correlation was observed between DF and CB (*r* = −0.18^**^ and *r* = −0.35^***^) and DPM and CB (*r* = −0.13^*^ and *r* = −0.20^**^) under irrigated and drought conditions, respectively. The PNA, SNA, and 100 seed weight showed a positive and highly significant correlation with grain yield under both irrigated and drought treatments (Table 3). The lines BFS 29, BFS 10, NCB 280, SEN 56, SCR 16, SCR 2, and NCB 226 showed higher values of PNA than the other genotypes under drought stress conditions (data not shown). These lines also presented higher values of SNA. The line SEA 15 showed the highest value of 100 seed weight under drought stress followed by NCB 226, SCR 2, SCR 16, NCB 280, and BFS 10. The genotypes ALB 88, DOR 390, SMC 43, Pérola, and MIB 778 produced less pods and seeds under drought stress.

## Discussion

This study permitted evaluating a diverse set of elite common bean breeding lines recently developed for improving resistance to drought stress. It was conducted in the light of past experience with shoot traits such as CID, stomatal conductance, canopy biomass, and indices of dry matter partitioning such as PPI, PHI, and HI. The lines were derived from crosses among bean races (Beebe et al., 2008), as well as interspecific crosses with introgression from *P. coccineus* (Butare et al., 2012) or *P. acutifolius* (Beebe, 2012). The lines NCB 280, NCB 226, SEN 56, SCR 2, SCR 16, SMC 141, RCB 593, BFS 67, SER 16, ALB 60, ALB 6, BFS 10, and BFS 29 developed over several cycles of breeding to drought limitation were found to be remarkably drought resistant. These lines also showed values of grain yield that doubles the values under drought stress of three leading commercial cultivars in Latin America: DOR 390, Perola, and Tío Canela. We removed the effects of drought escape (early flowering) and yield potential (optimally irrigated yield) by estimating DRI through analysis. We found a relationship between genotypes with higher values of grain yield under drought stress and higher values of DRI. This positive relationship indicates that genotypes with higher values of grain yield under drought stress are physiologically responsive to drought stress. This analysis also allowed us to examine drought resistance that can be explained by physiological measurements made in this study and discussed below.

### Grain yield and carbon isotope discrimination (CID)

**Figure 1:**
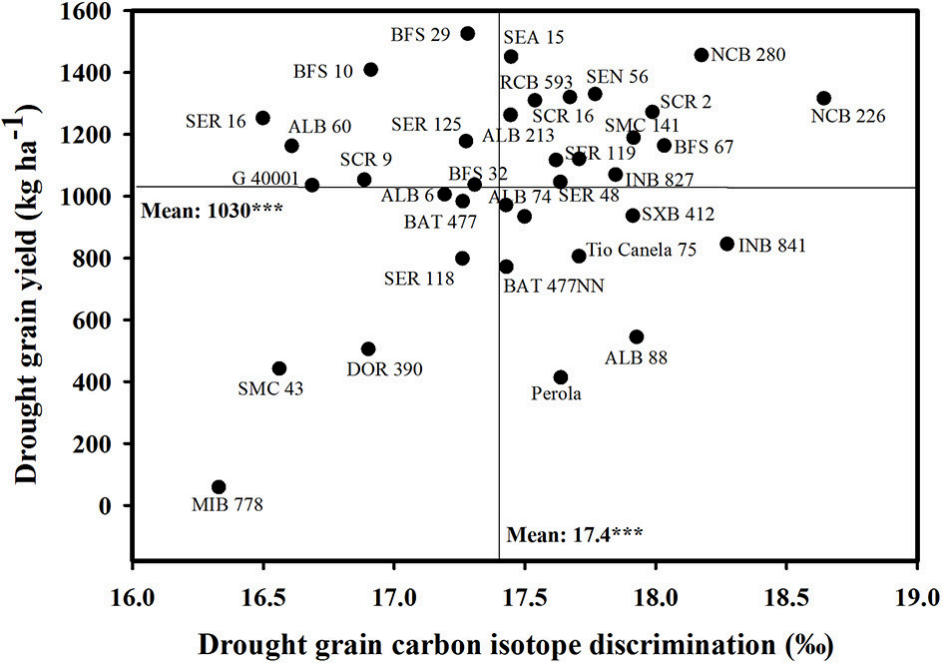
**Relationship between grain yield and grain carbon isotope discrimination (CID) under drought stress**. Mean values for two seasons (2012, 2013) were used. Water spenders with higher grain yield and greater values of CID were identified in the upper, right hand quadrant. Water savers with higher grain yield and lower values of CID were identified in the upper, left hand quadrant.

The relationship between different tissues sampled for CID and grain yield, showed that using grain sample is more relevant than CB sample at mid-pod filling in the determination of CID for common bean under drought conditions. We consider that the use of grain sample for CID determination makes more sense in the case of common bean under terminal drought stress. This is because it is taken at maturity, and it would reflect an integrated effect of gas exchange during a critical and important crop growth stage which is grain filling. In our experimental conditions the effect of terminal drought is stronger at harvest than at mid-pod filling. A relationship was observed between stomatal conductance and grain CID under rainfed conditions, with differences in performance among some genotypes (Figure 1, Table 2). Stomatal conductance and CID are useful measurements to track plant responses to drought but these depend on different processes. CID depends on variation in photosynthetic biochemistry, conductance of CO_2_ to the leaf interior and the chloroplasts, or a combination of these (Seibt et al., 2008; Easlon et al., 2014). After evaluating 36 advanced lines over two seasons, several drought resistant genotypes showed a clear evidence of superior access to soil moisture, and a few drought resistant genotypes showed a contrasting pattern for using less water. These results allowed us to classify the genotypes into two groups: water savers (isohydric plants) and water spenders (anisohydric) and this could facilitate targeting genotypes suitable to different agro-ecological zones (Blum, 2015). We classify NCB 280, SMC 141, SCR 16, SEN 56, BFS 67, and NCB 226 as water spenders that are able to access more water and show better water status during their canopy growth and grain development, resulting in higher grain yield. Our results also indicate that these lines maximize their capture of soil water for transpiration and maintain adequate gas exchange rates thus contributing to improved growth and greater proportion of biomass partitioned to grain (Figure 1, Tables 1, 2). We consider that these lines were drought resistant through EUW as proposed by Blum (2009). These genotypes should be useful for cultivation in areas exposed to intermittent drought stress in Central America, South America, and Africa, particularly in agro-ecological regions where rainfall is intermittent during the season and soils that can store greater amount of available water deep in the soil profile.

We also identified a few genotypes that are combining lower values of grain CID and stomatal conductance with better grain yield under rainfed conditions, and we classified these genotypes as water savers, which are BFS 10, SER 16, ALB 6, ALB 60, and G 40001 (Figure 1, Tables 1, 2). The line G 40001 (*P. acutifolius*) of tepary bean presents different traits related to drought resistance confirming its behavior as water saver with a combination of morpho-physiological characteristics such as greater ability for partitioning dry matter to grain, fine roots, smaller leaves for reduced water use, and reduced stomatal conductance (Mohamed et al., 2005; Butare et al., 2011; Rao et al., 2013; Beebe et al., 2014). Our classification of the line SER 16 as water saver is consistent with a previous study conducted under greenhouse conditions where this line was characterized as responsive to soil drying by closing its stomata sooner than the other genotypes during progressive soil drying (Devi et al., 2013). The water savers could be more suitable to bean farmers in semiarid to dry environments, dominated by terminal type of drought stress in Central America, Africa, northern Mexico, and north-east Brazil.

We also identified a group of genotypes (BFS 29, SEA 15, SER 125, and RCB 593) with intermediate response in terms of either as water spenders or as water savers with higher grain yield and these genotypes may be suitable for improving yield stability in drought-prone areas. The correlation between grain CID and grain yield under drought stress was weak but positive (Table 3). This is because of a few water saving genotypes that influenced the correlation. The values of grain CID are useful to classify genotypes as either water spenders or water savers together with other morpho-physiological attributes such as leaf stomatal conductance.

### Grain yield, canopy biomass, and indices of dry matter partitioning

**Figure 2:**
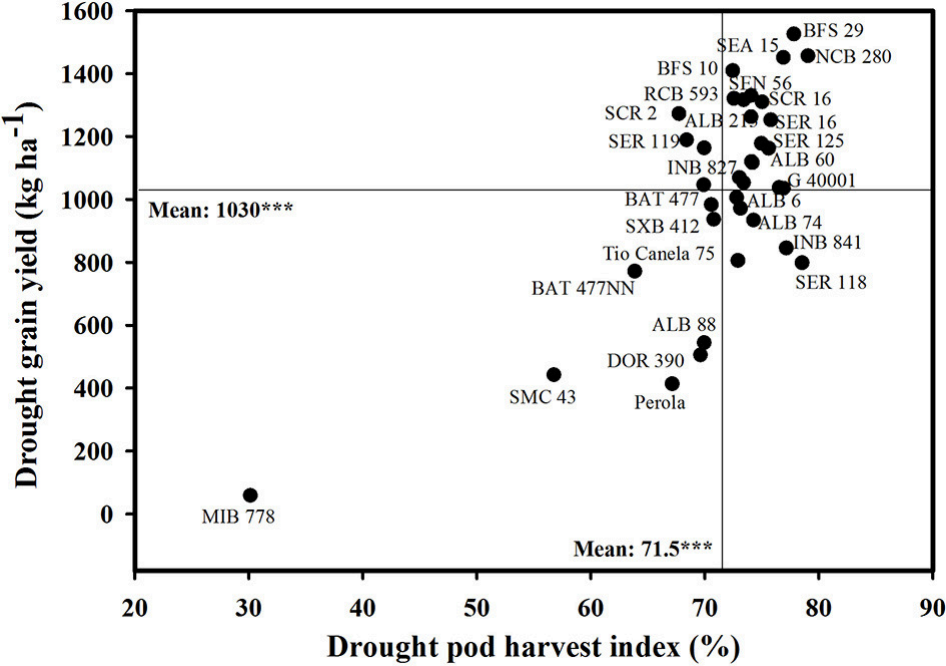
**Identification of genotypes with greater values of grain yield and pod harvest index (PHI) under drought stress**. Mean values for two seasons (2012, 2013) were used. Higher yielding genotypes with greater values of PHI were identified in the upper, right hand quadrant.

Total shoot biomass or CB can be understood in a physiological sense as the result of accumulated net photosynthesis of the crop, and it is shown to be related to yield in several crops (Araus et al., 2002). Several drought resistant lines were outstanding in CB production and grain yield under drought stress. Also these lines were superior in leaf area development under drought stress. These lines were able to access more water, as reflected in the values of grain CID. This, combined with increased dry matter partitioned to grain (HI and PHI), resulted in improved resistance to drought (Muñoz-Perea et al., 2007; Polania et al., 2012, 2016; Assefa et al., 2013; Rao et al., 2013; Beebe et al., 2014; Rao, 2014). Compared with the drought-sensitive checks DOR 390, Pérola, and Tio Canela, most of the bred lines showed a higher accumulation of CB at mid-pod filling growth stage. These results show significant progress in breeding for improved CB accumulation and enhanced dry matter partitioning toward pod and grain production. Previous research suggested that the drought resistance in common bean is associated with a more efficient dry matter partitioning to pod formation and grain production (Hall, 2004; Rosales-Serna et al., 2004; Cuellar-Ortiz et al., 2008; Klaedtke et al., 2012; Rosales et al., 2012; Assefa et al., 2013; Beebe et al., 2013, 2014; Rao et al., 2013; Rao, 2014). The positive and significant correlations between grain yield and biomass partitioning indices (PPI, HI, and PHI) under drought stress (Table 3) highlight the importance of improved dry matter partitioning from plant biomass to pod formation (PPI) and grain production (PHI). Genotypes that increased the proportion of dry matter partitioned to grain also showed higher values of TNC in seed (results not shown). The lines BFS 29, SEA 15, BFS 10, NCB 280, SEN 56, SCR 16, and SMC 141 showed greater grain yield under drought and were superior in their ability to partition greater proportion of biomass to pod and grain production (Figure 2, Table 1). The lines INB 841 and SER 118 showed especially higher values of PPI and PHI across years under stress, and both are excellent parents. Breeding programs should focus on the selection of best-performing materials that combine greater values of CB as a result of EUW with greater ability to partition dry matter toward pod development and grain filling. PHI could serve as a useful selection criterion for improving drought resistance because of its simplicity in measurement and its significant correlation with grain yield under both irrigated and drought conditions (Assefa et al., 2013).

### Grain yield, phenology, and yield components

Early maturing genotypes were more adapted to drought stress and responsive to irrigation. Farmers have multiple reasons for preferring short season varieties, one of them is to minimize exposure to drought (White and Singh, 1991; Beebe, 2012). Early maturity has been a standard mechanism to confront drought in breeding programs, and it is a trait that may be more useful where terminal drought predominates (Beebe et al., 2014). However, previous results indicated that a shorter growth cycle can reduce grain yield potential per day by an estimated 74 kg ha^−1^ (White and Singh, 1991). In contrast, recent results show that early maturing genotypes with increased dry matter partitioned to grain can compensate this effect, indicating that high yielding lines had higher grain yield per day compared with low yielding genotypes under drought (Klaedtke et al., 2012; Rao et al., 2013; Beebe et al., 2014). We also observed a negative and significant correlation between phenology (DF and DPM) and CB and phenology and SNA under both irrigated and drought stress conditions. These phenotypic correlations suggest that a rapid accumulation of CB due to EUW, combined with an efficient remobilization of these reserves to the pod and grain formation (higher values of grain yield per day and SNA), is an important adaptive strategy of early maturing and drought resistant genotypes. Significant positive associations between grain yield and PPI, PHI and 100 seed weight under both irrigated and drought conditions suggest that increased dry matter partitioning toward grain, or sink strength, is an important factor in determining grain yield under drought stress (Polania et al., 2016). Greater values of SNA and PNA likewise are consistent with the hypothesis that drought resistant lines have greater sink strength.

An important role for EUW in drought resistance implies that the understanding of the factors controlling the deep rooting and water status of the plant would be of great importance to improve drought resistance. In this scenario, traits such as grain CID related with EUW, CB, PPI, PHI, PNA, and SNA should be also considered as useful plant traits to be included in bean breeding programs aimed for improving drought resistance. Some of these traits are easier to implement in a breeding program due to their simplicity and relatively low analytical cost: PHI, grain CID, PNA, and SNA. Since these parameters could be determined at harvest time, it may be easier for breeders to integrate into on-going breeding efforts.

## Conclusions

Our results demonstrate that drought resistance in common bean is related to EUW to produce greater canopy biomass, combined with an increased dry matter partitioned from vegetative structures to the pods and subsequently to grain production resulting in higher values of pod and SNA. Several lines (BFS 29, SEA 15, BFS 10, NCB 280, SEN 56, SCR 16, and SMC 141) expressed this desirable combination of traits. Resistance to terminal drought stress was found to be positively associated with EUW combined with superior ability to partition greater proportion of dry matter toward pod and seed production and negatively associated with days to flowering and DPM. Based on phenotypic differences in grain CID, leaf stomatal conductance, canopy biomass and grain yield under drought stress, the lines tested were classified into two groups, water savers and water spenders. We suggest that pod harvest index could be a useful selection criterion in breeding programs to select for drought resistance in common bean.

## Author contributions

JP, CP, SB, and IR designed the experiments and contributed to data interpretation. JP collected and analyzed the data. JP, CP, SB, and IR wrote the paper. All authors read and approved the final manuscript.

### Conflict of interest statement

The authors declare that the research was conducted in the absence of any commercial or financial relationships that could be construed as a potential conflict of interest.
